# Assessment of the Tumor Redox Status in Head and Neck Cancer by ^62^Cu-ATSM PET

**DOI:** 10.1371/journal.pone.0155635

**Published:** 2016-05-17

**Authors:** Tetsuya Tsujikawa, Satoko Asahi, Myungmi Oh, Yoshitaka Sato, Norihiko Narita, Akira Makino, Tetsuya Mori, Yasushi Kiyono, Tatsuro Tsuchida, Hirohiko Kimura, Shigeharu Fujieda, Hidehiko Okazawa

**Affiliations:** 1 Biomedical Imaging Research Center, University of Fukui, Fukui, Japan; 2 Department of Radiology, Faculty of Medical Sciences, University of Fukui, Fukui, Japan; 3 Department of Otolaryngology, Faculty of Medical Sciences, University of Fukui, Fukui, Japan; Wayne State University, UNITED STATES

## Abstract

**Purpose:**

Tumor redox is an important factor for cancer progression, resistance to treatments, and a poor prognosis. The aim of the present study was to define tumor redox (over-reduction) using ^62^Cu-diacetyl-bis(*N*^4^-methylthiosemicarbazone) (^62^Cu-ATSM) PET and compare its prognostic potential in head and neck cancer (HNC) with that of 2-deoxy-2-[^18^F]fluoro-D-glucose (^18^F-FDG).

**Methods:**

Thirty HNC patients (stage II–IV) underwent pretreatment ^62^Cu-ATSM and ^18^F-FDG PET scans. Maximum standardized uptake values (SUV_ATSM_ and SUV_FDG_) and tumor-to-muscle activity concentration ratios (TMR_ATSM_ and TMR_FDG_) were measured. Reductive-tumor-volume (RTV) was then determined at four thresholds (40%, 50%, 60%, and 70% SUV_ATSM_), and total-lesion-reduction (TLR) was calculated as the product of the mean SUV and RTV for ^62^Cu-ATSM. In ^18^F-FDG, metabolic-tumor-volume (MTV) and total-lesion-glycolysis (TLG) were obtained at a threshold of 40%. A ROC analysis was performed to determine % thresholds for RTV and TLR showing the best predictive performance, and these were then used to determine the optimal cut-off values to stratify patients for each parameter. Progression-free-survival (PFS) and cause-specific-survival (CSS) were evaluated by the Kaplan-Meier method.

**Results:**

The means ± standard deviations of PFS and CSS periods were 16.4±13.4 and 19.2±12.4 months, respectively. A ROC analysis determined that the 70% SUV_ATSM_ threshold for RTV and TLR was the best for predicting disease progression and cancer death. Optimal cut-offs for each index were SUV_ATSM_ = 3.6, SUV_FDG_ = 7.9, TMR_ATSM_ = 3.2, TMR_FDG_ = 5.6, RTV = 2.9, MTV = 8.1, TLR = 14.0, and TLG = 36.5. When the cut-offs for TMR_ATSM_ and TLR were set as described above in ^62^Cu-ATSM PET, patients with higher TMR_ATSM_ (p = 0.03) and greater TLR (p = 0.02) showed significantly worse PFS, while patients with greater TLR had significantly worse CSS (p = 0.02). Only MTV in ^18^F-FDG PET predicted differences in PSF and CSS (p = 0.03 and p = 0.03, respectively).

**Conclusion:**

Tumor redox parameters measured by ^62^Cu-ATSM PET may be determinants of HNC patient outcomes and help define optimal patient-specific treatments.

## Introduction

**Fig 1:**
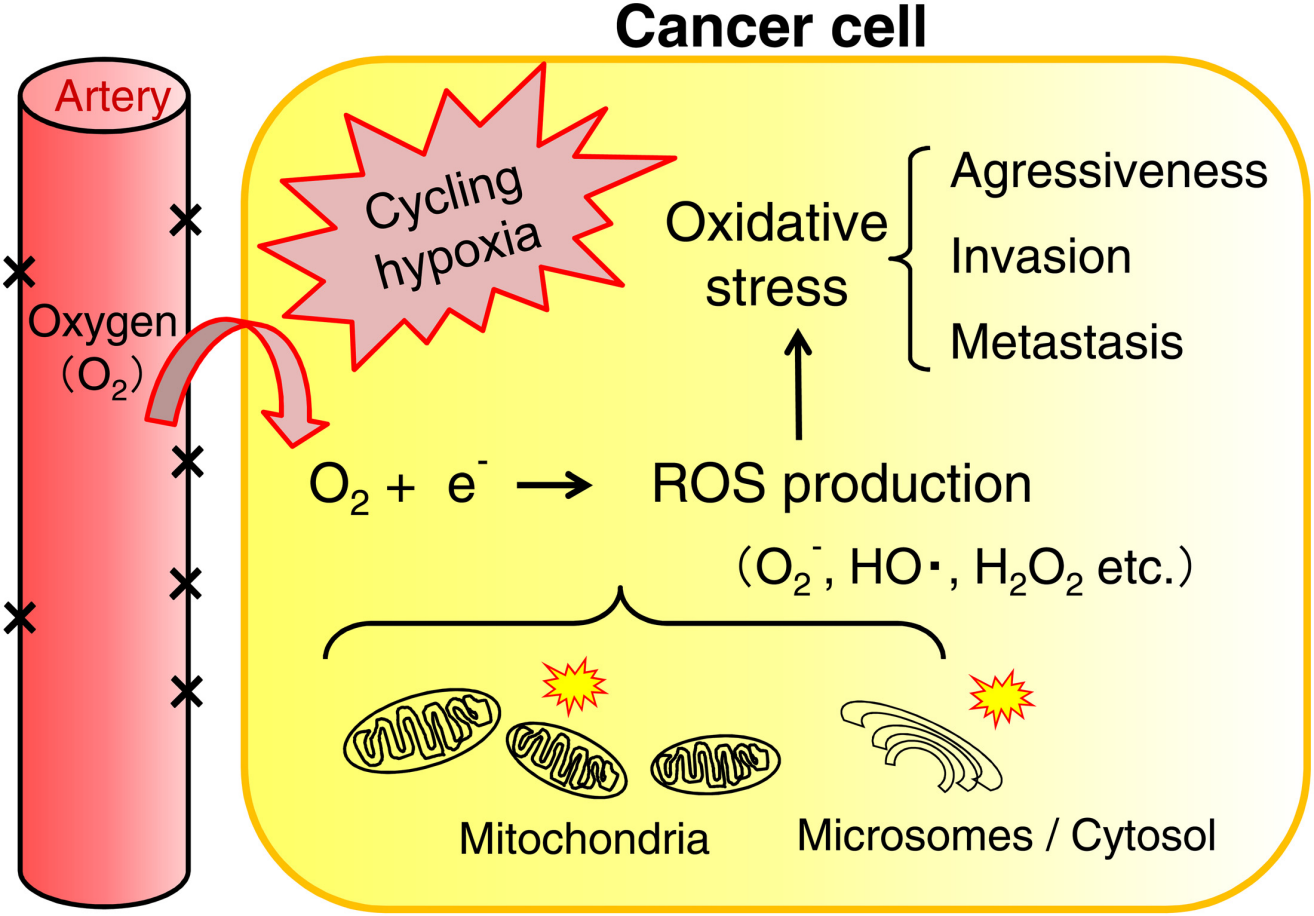
Oxidative stress in cancer cells under cycling hypoxic conditions. Oxidative stress is a state of redox imbalance caused by the increased production of reactive oxygen species (ROS), which are mostly generated by the leakage of excessive levels of electrons relative to O_2_ in impaired mitochondrial respiratory chains. ROS damage proteins and DNA/RNA and also act as signaling molecules to drive cancer cell motility/invasion and tumor progression. ROS (superoxide anion: O_2_-, hydrogen radical: HO., hydrogen peroxide: H_2_O_2_)

The tumor microenvironment is characterized by hypoxia (a low partial pressure of oxygen: a low pO_2_) and a highly reducing redox status [1–3]. Cancer cells are capable of surviving under hypoxic conditions by inducing the expression of metabolic enzymes required for anaerobic metabolism such as glycolysis. They may also induce the formation of blood vessels by a process called angiogenesis in order to fulfill their oxygen and nutritional requirements. However, new blood vessels are often poorly formed, thereby leading to an unstable cancer environment that oscillates between low and moderate to high oxygen conditions. This cycling phenomenon is termed cycling hypoxia. An over-reductive state is the state in which cancer cells and tissues contain excessive levels of electrons relative to O_2_, which is caused by impaired respiratory chains or hypoxia, and oxidative stress is induced by excess reactive oxygen species (ROS) produced from O_2_ and redundant electrons due to mitochondrial dysfunction. Evidence to suggest the importance of the redox status for cancer progression, resistance to treatments, and a poor prognosis is mounting [4–10] (Fig 1).

**Fig 2:**
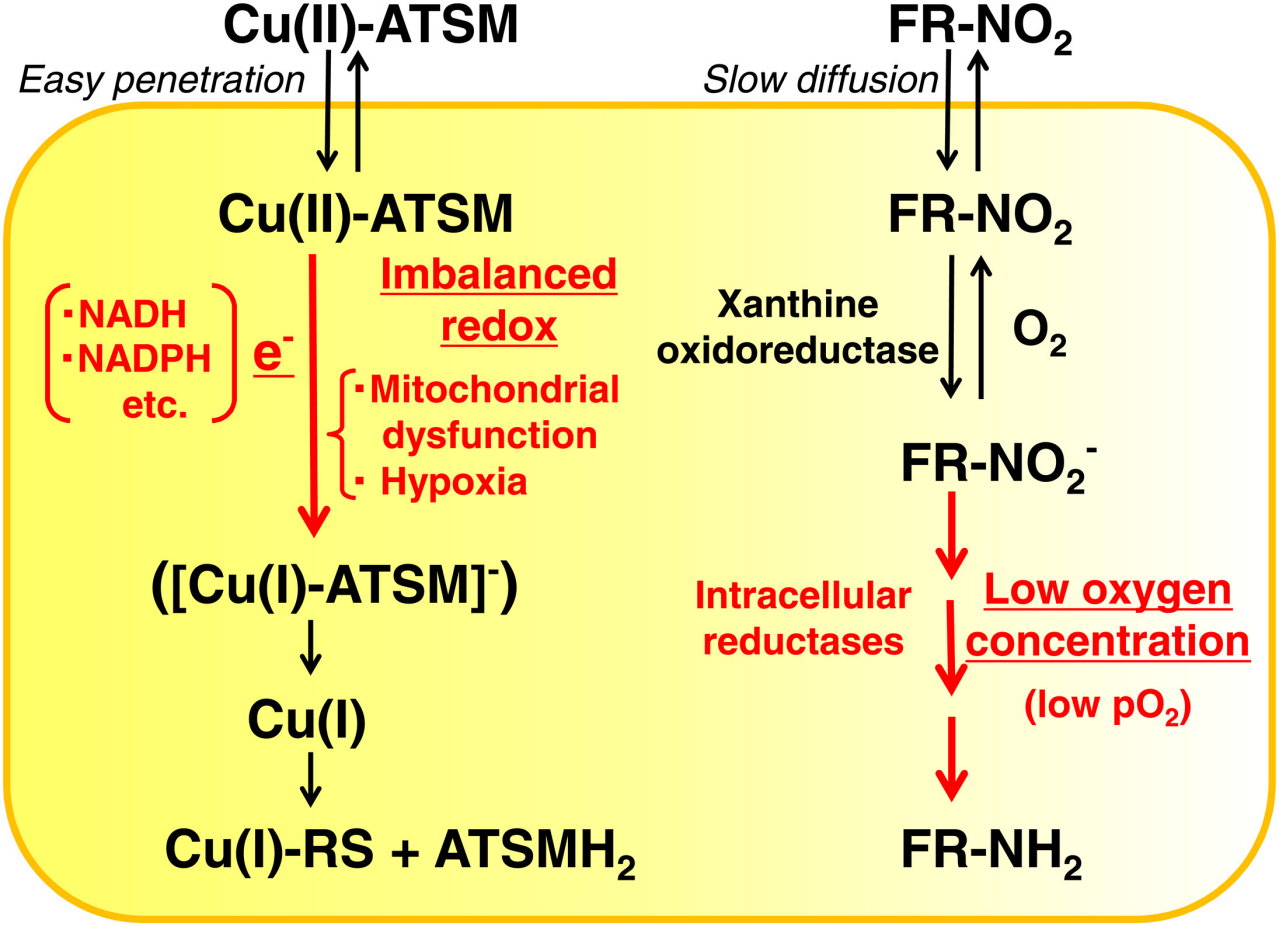
Cu(II)-ATSM and fluorinated nitroimidazole (FR-NO_2_) retention mechanisms in cancer cells. During the course of tracer retention in cancer cells, key factors are shown in red for both Cu(II)-ATSM and fluorinated nitroimidazole (FR-NO_2_). Cu(II)-ATSM is a neutral lipophilic molecule that easily penetrates cell membranes. In cancer cells over-reduced due to mitochondrial dysfunction and hypoxia, Cu(II)-ATSM may be converted to [Cu(I)-ATSM]^-^ with electrons (e^-^) supplied from abnormally reduced mitochondria in a number of forms including NADH and NADPH, and retained in cells because of its negative charge. Cu(I) is subsequently dissociated by reactive chemical species (RS) generated in the reduced condition and is irreversibly trapped as Cu(I)-RS in cells. FR-NO_2_ pass through cell membranes by slow diffusion and may be converted to a reduced form, FR-NO_2_^-^, by xanthine oxidoreductase. Under hypoxic conditions (low pO_2_), FR-NO_2_^-^ may be reduced further by intracellular reductases in a low oxygen concentration-dependent manner to R-NH_2_, which binds covalently to macromolecules in cancer cells.

In order to measure the tumor redox status *in vivo*, magnetic resonance imaging (MRI) with redox-sensitive contrast agents has been performed on animal models [11, 12]. Copper(II) diacetyl-bis(*N*^4^-methylthiosemicarbazone) (Cu-ATSM) is one of the two main hypoxia-seeking ligands for positron emission tomography (PET), with the other being fluorinated 2’-nitroimidazoles represented by ^18^F-fluoromisonidazole (^18^F-FMISO) [13, 14]. Previous studies reported that a good correlation existed between low pO_2_ directly measured by polarographic oxygen electrodes and high tracer accumulation *in vivo* [15, 16]. However, discrepancies have been identified between the distributions of Cu-ATSM and FMISO in different tumor tissue types [17, 18]. Although the Cu(II)-ATSM retention mechanism is not yet fully understood [19–22], Cu(II)-ATSM PET may be reassessed as tumor over-reduction imaging that is distinctive from PET with fluorinated nitroimidazoles (FR-NO_2_) (Fig 2). Cu(II)-ATSM is a neutral lipophilic molecule that easily penetrates cell membranes. In cancer cells over-reduced due to mitochondrial dysfunction and hypoxia, Cu(II)-ATSM may be converted to [Cu(I)-ATSM]^-^ with electrons (e^-^) supplied from abnormally reduced mitochondria in a number of forms including NADH and NADPH, and retained in cells because of its negative charge. Cu(I) is subsequently dissociated by reactive chemical species (RS) generated in the reduced condition and is irreversibly trapped as Cu(I)-RS in cells. FR-NO_2_ passes through cell membranes by slow diffusion and may be converted to a reduced form, FR-NO_2_^-^, by xanthine oxidoreductase [19]. Under hypoxic conditions (low pO_2_), FR-NO_2_^-^ may be reduced further by intracellular reductases in a low oxygen concentration-dependent manner to R-NH_2_, which binds covalently to macromolecules in cancer cells. The accumulation of FR-NO_2_ is subject to a low oxygen concentration, while Cu-ATSM uptake is assumed to reflect the imbalanced redox (over-reductive) status in cancer cells. We herein defined a new concept of ‘tumor redox imaging with Cu-ATSM PET’, which means Cu-ATSM may be used distinctively from FR-NO_2_ in order to determine the imbalanced redox (over-reductive) status in cancer cells, which is important for appropriate treatment strategies and prognoses.

Glycolysis is generally accelerated in cancer cells even in the presence of oxygen (the Warburg effect), which enables 2-deoxy-2-[^18^F]fluoro-D-glucose (^18^F-FDG) PET to determine the cancer stage, monitor treatment responses, and predict long-term prognoses. The predictive value of ^18^F-FDG PET for the prognoses of patients with head and neck cancer (HNC) has recently been reported, particularly using volume-based metabolic parameters such as metabolic-tumor-volume (MTV) and total-lesion-glycolysis (TLG) [23–25]. We recently reported that the intensity-based parameters of ^62^Cu-ATSM PET (standardized uptake value: SUV and tumor-to-muscle activity ratio: TMR) may be better prognostic markers than those of ^18^F-FDG PET in HNC [26]. On a ^62^Cu-ATSM PET intensity basis, HNC patients with the higher tumor accumulation of ^62^Cu-ATSM had significantly worse prognoses than those with low-uptake tumors. In contrast, no significant difference was observed in prognoses between HNC patient groups showing higher and lower ^18^F-FDG uptakes.

The aim of the present study was to investigate the predictive performance of ^62^Cu-ATSM and ^18^F-FDG PET-derived parameters of lesion image intensity and volume in HNC. To the best of our knowledge, no study has yet defined ^62^Cu-ATSM PET as over-reduction imaging in tumors or evaluated the value of volumetric PET parameters with ^62^Cu-ATSM reflecting a reductive tumor burden for prognostic predictions.

## Materials and Methods

### Patients

Thirty untreated patients (24 males, 6 females; 68.3 ± 12.4 years of age) with biopsy-proven stage II to IV HNC at the University of Fukui Hospital between April 2007 and October 2012 were enrolled in this prospective study (Table 1). The primary tumor sites were the oral cavity (*n* = 9), paranasal sinus (*n* = 5), oropharynx (*n* = 5), hypopharynx (*n* = 2), larynx (*n* = 3), salivary gland (*n* = 5), and other (*n* = 1). Histologies were 24 squamous cell carcinomas and 6 adenocarcinomas. All patients underwent CT and magnetic resonance imaging scans in order to obtain local information as well as whole-body ^18^F-FDG PET/CT scans for staging. Each patient underwent ^62^Cu-ATSM PET within a week of the ^18^F-FDG PET/CT scan. This study was approved by the Ethics Committee of the University of Fukui, Faculty of Medical Sciences. Written informed consent was obtained from all individual participants included in the study. In this prospective study, we increased the number of patients from that in our previous study with HNC and also extended follow-up periods [26].

**Table 1:** Patient Characteristics.

		Staging			Treatment				Response	
Organ	N	Stage II	Stage III	Stage IV	CRT + SO	CRT	SO + RT	SO	CR	Non CR
Oral cavity	9	1	6	2	5	4			5	4
Paranasal sinus	5			5	1	4			2	3
Oropharynx	5			5	3	2			2	3
Hypopharynx	2	1		1	2				0	2
Larynx	3	1		2		2		1	2	1
Salivary gland	5	1	1	3	3		1	1	4	1
Other	1			1				1	0	1
Total	30	4	7	19	14	12	1	3	15	15

CRT: chemoradiation therapy, SO: surgical operation, RT: radiation therapy, CR: complete response

### Preparation of ^62^Cu-ATSM

^62^Cu glycine (non-carrier added ^62^Cu) solution was obtained from a ^62^Zn/^62^Cu generator system every hour [27, 28]. ^62^Cu-ATSM was prepared with a simple mixture of ^62^Cu solution (5 mL) and 0.2 mL of ATSM solution (0.5 mM in dimethyl sulfoxide) in a sterilized vial [15]. The radiochemical purity of ^62^Cu-ATSM was confirmed with high-performance liquid chromatography (HPLC) using authentic unlabeled Cu-ATSM prior to the first injection administered to patients.

### PET Procedure

PET procedures were described in detail in our previous study [26]. Briefly, a whole-body PET scanner (Advance, General Electric Medical Systems, Milwaukee, WI) was used for ^62^Cu-ATSM PET studies and a 20-minute dynamic PET image acquisition, including known primary tumor sites, was performed after an intravenous injection of 600 to 800 MBq (approximately 16–22 mCi) of ^62^Cu-ATSM over 30 seconds. The dynamic frames were 10 seconds × 12 frames, 60 seconds × 8 frames, and then 5 minutes × 2 frames. ^18^F-FDG PET images were acquired in the static mode with a whole-body PET/CT scanner (Discovery LS, General Electric Medical Systems, Milwaukee, WI) approximately 50 minutes after the administration of 185 MBq (5 mCi) of ^18^F-FDG. All patients fasted for at least 4 hours before the ^18^F-FDG PET study.

PET images of ^62^Cu-ATSM and ^18^F-FDG were reconstructed using the iterative reconstruction method with 14 subsets and 2 iterations with a spatial resolution of 6-mm full width at half maximum. The reconstructed images were converted to semiquantitative images parameterized in units of SUV.

### Image Analysis

The time-activity curves of ^62^Cu-ATSM PET obtained from dynamic data showed that all tumors had stable radiotracer retention by 8–10 minutes post-injection and in the later phase after the tracer injection [26–28]. Thus, an average image of the last 10-minute frame, which was considered appropriate scan timing for the tracer retention phase from time-activity curves (TACs), was used to evaluate the tumor redox status.

^62^Cu-ATSM and ^18^F-FDG PET images were co-registered based on their respective CT images from PET/CT using automatic registration software (AW VS4, GE Medical Systems, Milwaukee, WI). Volumes of interest (VOIs) were drawn on the primary tumor and bilateral sternocleidomastoid muscles. Regarding intensity-based parameters, the overall tumor uptake of ^62^Cu-ATSM was assessed semiquantitatively on late-phase images by determining the maximum SUV (SUV_ATSM_) and tumor-to-muscle activity concentration ratio (TMR_ATSM_) using tumor SUV_ATSM_ and muscle SUVs. The maximum SUV (SUV_FDG_) and tumor-to-muscle activity concentration ratio (TMR_FDG_) were determined in the same manner for ^18^F-FDG PET.

Tumor contours were delineated to include voxels presenting SUV values greater than 40%, 50%, 60%, and 70% SUV_ATSM_ for ^62^Cu-ATSM PET in order to determine optimal % thresholds for further analyses and 40% SUV_FDG_ for ^18^F-FDG PET. The threshold of 40% SUV_FDG_ for ^18^F-FDG PET was based on a previous study [29]. Tumor volumes were defined as reductive-tumor-volume (RTV) for ^62^Cu-ATSM and MTV for ^18^F-FDG. Total-lesion-reduction (TLR) was calculated as the product of average SUV and RTV for ^62^Cu-ATSM, and TLG was calculated as the product of the mean SUV and MTV for ^18^F-FDG.

### Statistical Analysis

In an attempt to determine whether the tumor uptake and volume of ^62^Cu-ATSM or ^18^F-FDG were predictive of treatment outcomes, a correlation analysis was performed between the results of PET and those of clinical follow-ups. The physician who assessed patients for disease progression and survival was blinded to the results of the ^62^Cu-ATSM and ^18^F-FDG PET studies.

A receiver-operating-characteristic (ROC) analysis was performed in order to determine the % thresholds for volume-based redox parameters (RTV and TLR) showing the best predictive performance based on the area under the curves (AUCs) and optimum cut-off values for each PET index in order to identify patients with or without events (disease progression and overall death) at the time of the last follow-up after the treatment. The significance of differences between the AUCs was tested using the pairwise comparison of DeLong et al. [30]. A ROC analysis and comparison of AUCs were performed using MedCalc^R^ (version 13.3.0.0; MedCalc Software bvba). The Kaplan-Meier method was used to assess the relationships between each PET parameter and progression-free survival (PFS) and cause-specific survival (CSS) rates. The equivalence of survival curves was tested with Log-rank (Mantel-Cox) statistics using GraphPad Prism^R^ (version 6.01; GraphPad Software, Inc.). A probability of less than 0.05 was considered significant.

## Results

### Patient Characteristics

The characteristics of all 30 patients are summarized in Table 1. Fourteen patients received chemoradiation therapy (CRT) and underwent surgical operations (SOs), twelve received CRT, one received radiation and underwent SO, and three underwent SO. Patients were clinically followed for periods ranging between 4 and 36 months (mean ± SD = 19.2 ± 12.4 months). At the last follow-up, 16 patients were alive for periods ranging between 7 and 36 months (mean ± SD = 27.6 ± 10.3 months): 13 with good control (complete response) and 3 with recurrence. Twelve patients died from local recurrence or metastasis of the primary cancer and 2 died from other diseases. The mean periods of PFS and CSS were 16.4 ± 13.4 months and 19.2 ± 12.4 months, respectively.

### Survival Prediction

Among the thresholds of 40%, 50%, 60%, and 70% SUV_ATSM_ for volume-based redox indices (RTV and TLR), ROC analyses showed that the AUCs of RTV_40%_, RTV_50%_, RTV_60%_, and RTV_70%_ were 0.56, 0.55, 0.56, and 0.64 for predicting disease progression and 0.58, 0.57, 0.58, and 0.68 for cancer death, respectively. Similarly, the AUCs of TLR_40%_, TLR _50%_, TLR _60%_, and TLR _70%_ were 0.61, 0.62, 0.60, and 0.65 for predicting disease progression and 0.55, 0.56, 0.57, and 0.64 for cancer death, respectively. Although no significant difference was observed among AUCs, we selected a threshold of 70% SUV_ATSM_ as the optimum threshold value parameter for predicting disease progression and cancer death because it yielded the largest AUCs.

In all 30 patients, SUV_ATSM_ (mean ± SD) was 4.1 ± 1.9 (g/ml), SUV_FDG_ was 10.8 ± 5.2 (g/ml), TMR_ATSM_ was 4.0 ± 1.8, TMR_FDG_ was 9.2 ± 4.7, RTV was 4.1 ± 3.8 (ml), MTV was 16.8 ± 15.7 (ml), TLR was 13.2 ± 11.8 (g), and TLG was 130.5 ± 181.5 (g).

**Fig 3:**
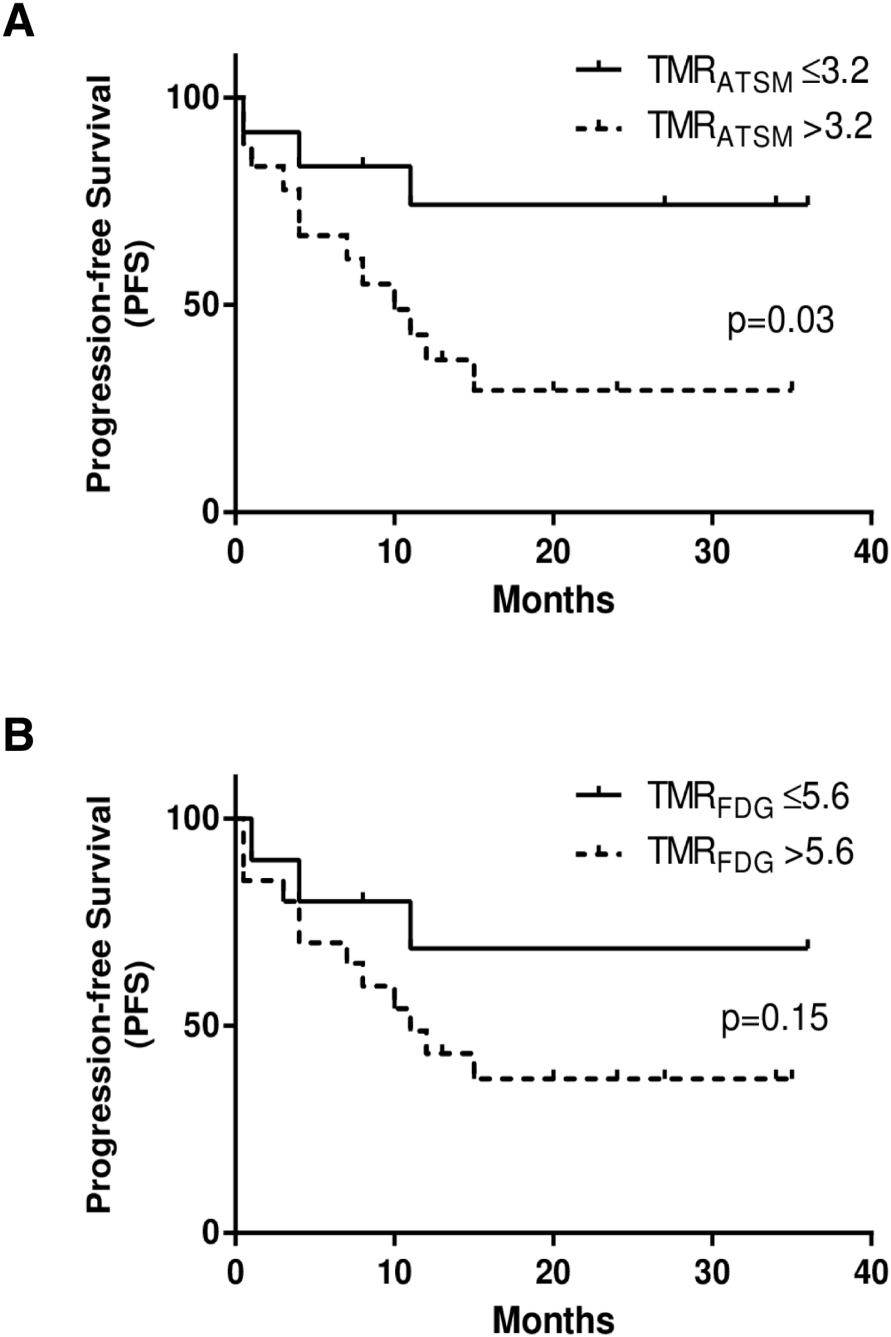
Kaplan-Meier curves of progression-free survival (PFS) for ^62^Cu-ATSM PET (a) and ^18^F-FDG PET (b) in patients with HNC. Two groups of high (dotted lines) and low (solid lines) tracer accumulation were determined by each cut-off value of the tumor-to-muscle ratios (TMR_ATSM_ and TMR_FDG_). TMR_ATSM_, one of the intensity-based redox parameters, showed a significant difference in PFS between two groups (*p* = 0.03), whereas TMR_FDG_, one of the intensity-based metabolic parameters, did not (*p* = 0.15). The three-year PFS rate was 74% for patients with lower accumulation tumors (TMR_ATSM_ ≤ 3.2) and 29% for those with over-reductive tumors (TMR_ATSM_ > 3.2).

**Fig 4:**
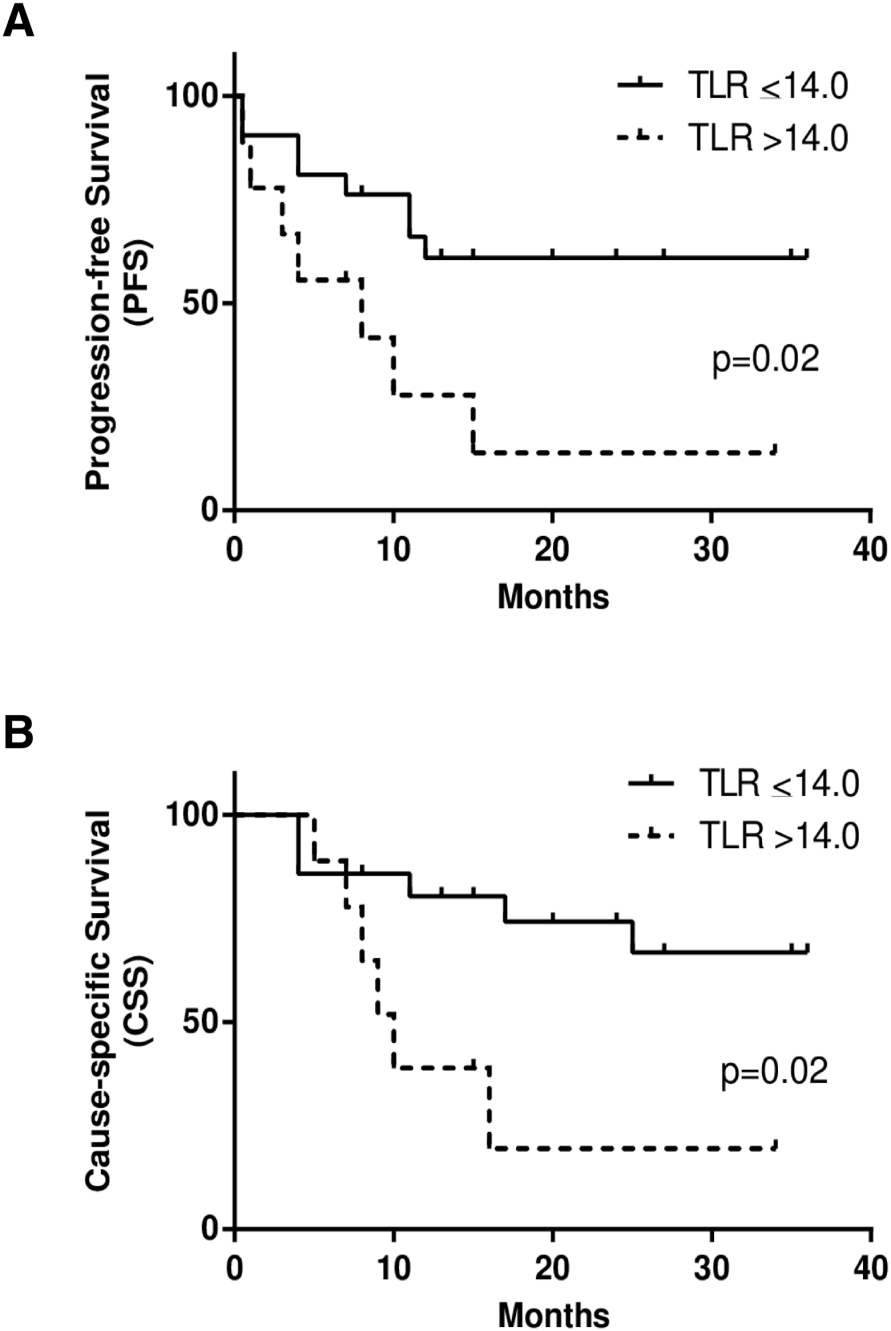
Kaplan-Meier curves of progression-free survival (PFS) (a) and cause-specific survival (CSS) (b) in patients with HNC. Two groups with the accumulation of large (> 14.0, dotted lines) and small (≤ 14.0, solid lines) amounts of ^62^Cu-ATSM were determined by total-lesion-reduction (TLR), one of the volume-based redox parameters. The two groups showed significant differences in PFS (*p* = 0.02) and CSS (*p* = 0.02). Three-year PFS and CSS rates were 61% and 67% for patients with a smaller reductive tumor burden (TLR ≤ 14.0), and 14% and 20% for those with a greater reductive tumor burden (TLR > 14.0), respectively.

**Fig 5:**
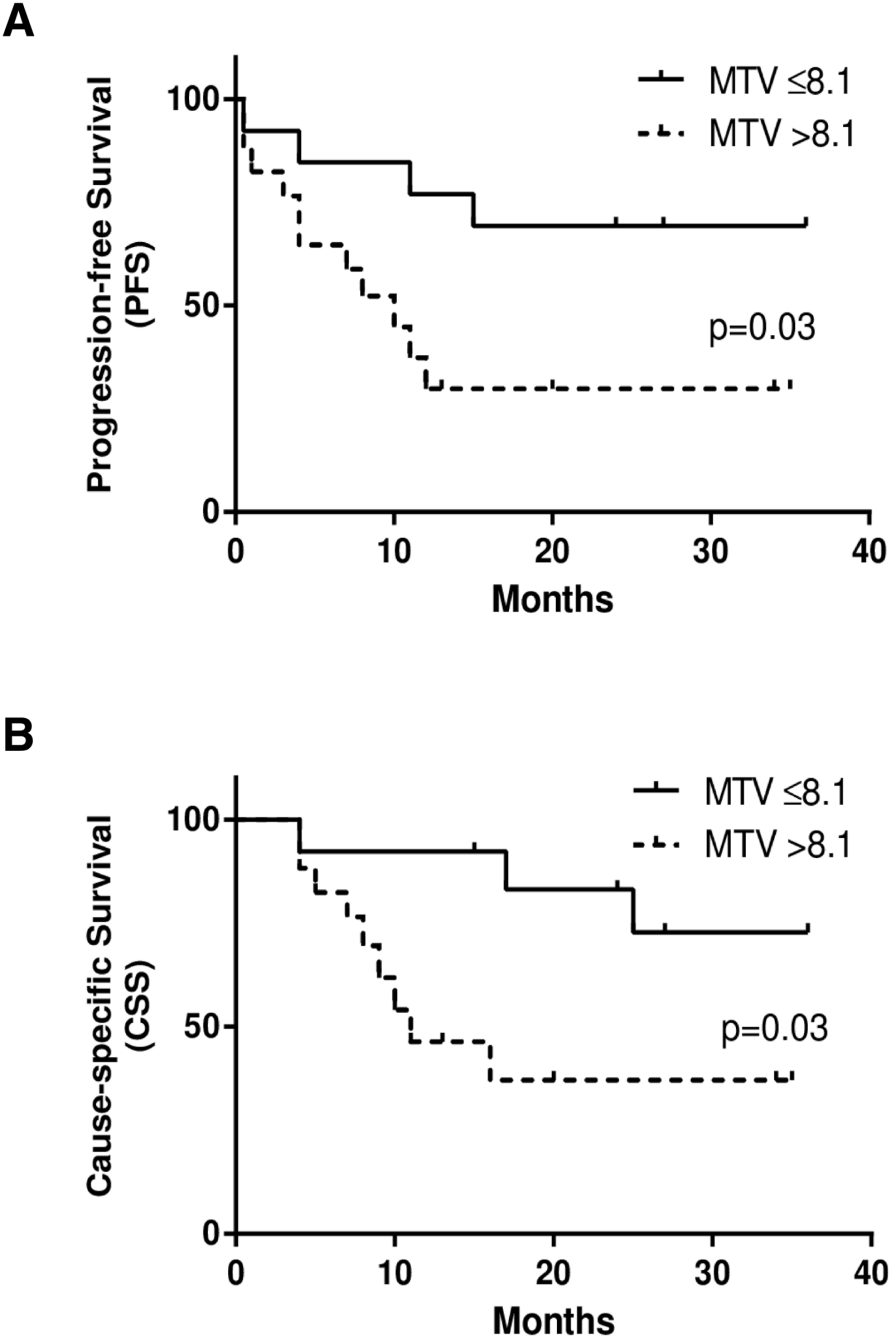
Kaplan-Meier curves of progression-free survival (PFS) (a) and cause-specific survival (CSS) (b) in patients with HNC. Two groups with the accumulation of large (> 8.1, dotted lines) and small (≤ 8.1, solid lines) volumes of ^18^F-FDG were determined by metabolic-tumor-volume (MTV), one of the volume-based metabolic parameters. The two groups showed significant differences in PFS (*p* = 0.03) and CSS (*p* = 0.03). Three-year PFS and CSS rates were 70% and 73% for patients with a smaller metabolic volume (MTV ≤ 8.1), and 30% and 37% for those with a larger metabolic volume (MTV > 8.1), respectively.

Optimum cut-off values determined by the ROC analysis for each PET index to divide patients with or without events were as follows: SUV_ATSM_ = 3.6, SUV_FDG_ = 7.9, TMR_ATSM_ = 3.2, TMR_FDG_ = 5.6, RTV = 2.9, MTV = 8.1, TLR = 14.0, and TLG = 36.5. When the cut-off values for TMR_ATSM_ and TLR were set as described above for ^62^Cu-ATSM PET, patients with higher TMR_ATSM_ had significantly worse PFS (*p* = 0.03), while those with greater TLR had significantly worse PFS and CSS (*p* = 0.02 and *p* = 0.02, respectively) (Figs 3A, 4A and 4B). Only MTV in ^18^F-FDG PET predicted differences in PSF and CSS (*p* = 0.03 and *p* = 0.03, respectively) (Fig 5A and 5B). SUV_ATSM_, SUV_FDG_, TMR_FDG_ (Fig 3B), RTV, and TLG did not show significant differences in PFS or CSS between the two groups.

### Representative Cases

**Fig 6:**
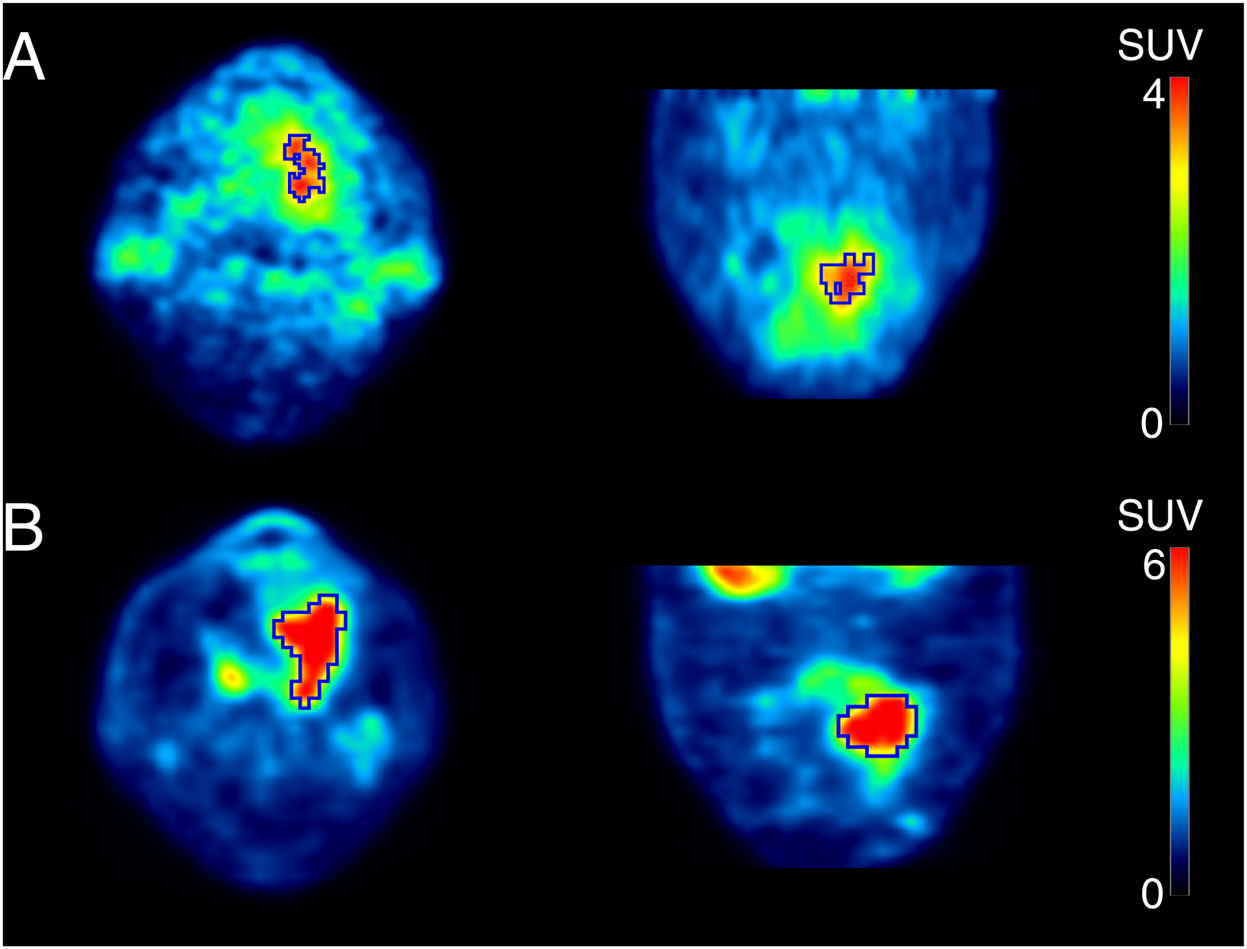
PET images of ^62^Cu-ATSM (a) and ^18^F-FDG (b) of a 62-year-old man with tongue cancer. Tumor contours were delineated to include voxels presenting SUV values greater than 70% SUV_ATSM_ of 4.6 for ^62^Cu-ATSM PET and 40% SUV_FDG_ of 10.1 for ^18^F-FDG PET. Volume-based parameters were calculated as follows; RTV = 3.6, MTV = 19.3, TLR = 12.8, and TLG = 115.9. He is still alive without any recurrence or metastasis after being treated (CRT + SO). The volume-based redox parameter, TLR, which was smaller than the cut-off value of 14.0, correctly predicted his outcome. On the other hand, volume-based metabolic indices were greater than each cut-off value.

Fig 6 shows a 62-year-old man with tongue cancer (SUV_ATSM_ = 4.6, SUV_FDG_ = 10.1). Using thresholds of 70% SUV_ATSM_ and 40% SUV_FDG_ to delineate tumor contours, volume-based redox and metabolic parameters were calculated as follows: RTV = 3.6, MTV = 19.3, TLR = 12.8, and TLG = 115.9. He is still alive without any recurrence or metastasis after being treated (CRT + SO). The volume-based redox parameter, TLR, which was smaller than the cut-off value, correctly predicted his outcome. On the other hand, volume-based metabolic indices were greater than each cut-off value.

**Fig 7:**
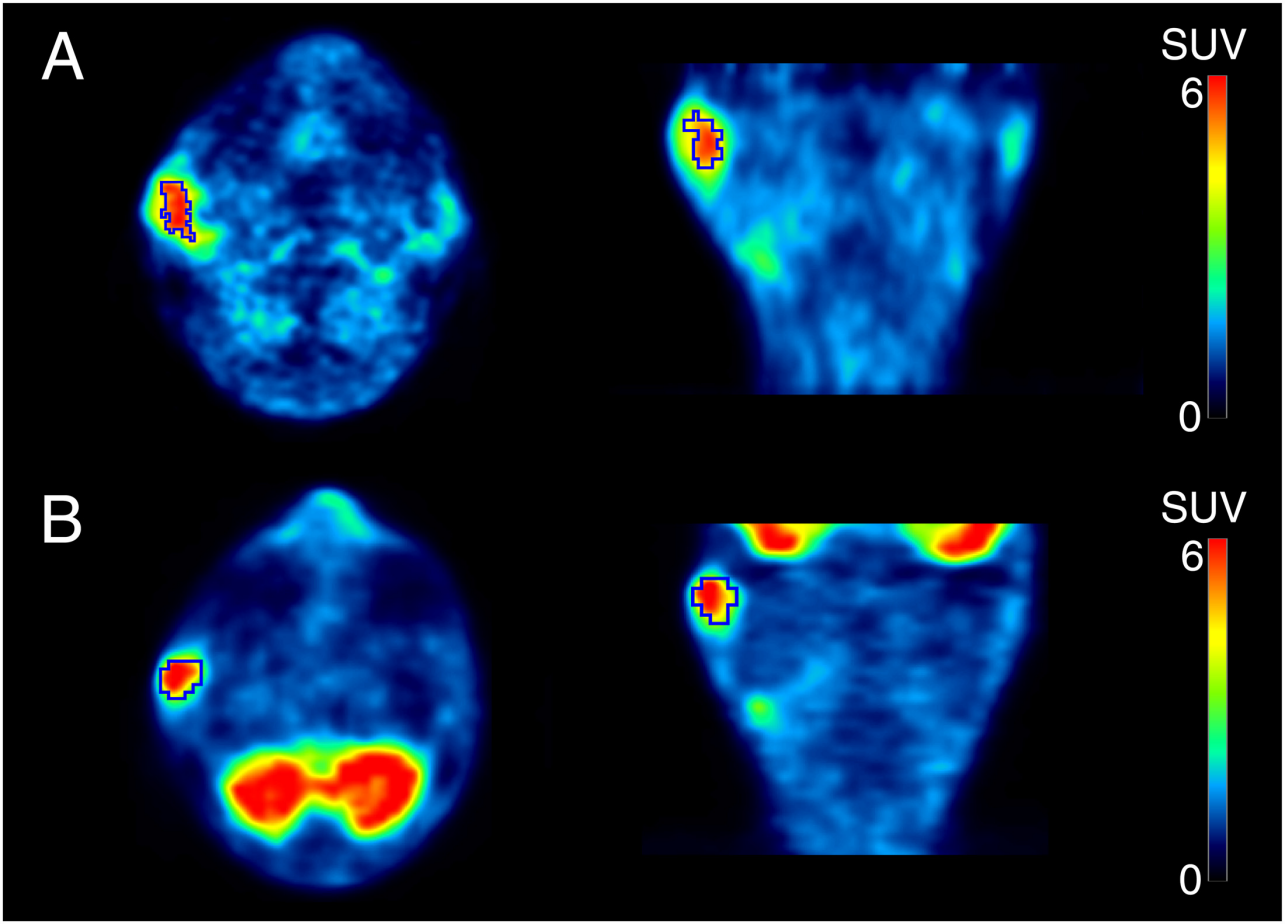
PET images of ^62^Cu-ATSM (a) and ^18^F-FDG (b) of a 64-year-old man with right parotid cancer. Tumor contours were delineated to include voxels presenting SUV values greater than 70% SUV_ATSM_ of 6.9 for ^62^Cu-ATSM PET and 40% SUV_FDG_ of 8.8 for ^18^F-FDG PET. Volume-based parameters were calculated as follows; RTV = 5.9, MTV = 6.3, TLR = 32.0, and TLG = 30.0. He developed iliac bone metastasis 15 months after being treated (CRT + SO). The volume-based redox parameters, RTV and TLR, which were greater than each cut-off value (RTV: 2.9 and TLR: 14.0, respectively), correctly predicted his outcome. On the other hand, volume-based metabolic indices were smaller than each cut-off value.

Fig 7 shows a 64-year-old man with right parotid cancer (SUV_ATSM_ = 6.9, SUV_FDG_ = 8.8). Using thresholds of 70% SUV_ATSM_ and 40% SUV_FDG_ to delineate tumor contours, volume-based redox and metabolic parameters were calculated as follows: RTV = 5.9, MTV = 6.3, TLR = 32.0, and TLG = 30.0. He developed iliac bone metastasis 15 months after being treated (CRT + SO). The volume-based redox parameters, RTV and TLR, which were greater than each cut-off value, correctly predicted his outcome. On the other hand, volume-based metabolic indices were smaller than each cut-off value.

## Discussion

High tumor uptakes of ^62^Cu-ATSM (TLR) as well as a high intensity (TMR_ATSM_) predicted poor prognoses in HNC patients, as determined by PFS and CSS. These results indicate the importance of detecting tumor over-reductive states, namely, a reductive intensity and reductive tumor burden for the biological characterization of tumors and survival prediction. ^62^Cu-ATSM PET imaging of an over-reductive state has potential as a marker for oxidative stress induced by the excessive production of ROS in cancer cells; it may provide information on the degree and amount of ROS in tumors, which drive cancer cell motility, invasion, tumor progression, and treatment resistance. We previously demonstrated that Cu-ATSM accumulated in regions rich in cancer cells expressing CD133, which is a frequently used marker for cancer stem cells or cancer stem cell-like cells [31], and a relationship has since been suggested to exist between these cells and poorer patient outcomes. Regarding metabolic PET parameters with ^18^F-FDG in the present study, the predictive performance of MTV for HNC patient outcomes was good, whereas intensity-based metabolic parameters (SUV_FDG_ and TMR_FDG_) had a poor predictive value. Information obtained on oxidative stress in tumors by ^62^Cu-ATSM PET may provide more accurate predictions of patient outcomes than glucose metabolism by ^18^F-FDG PET. In any case, since cycling hypoxia, redox, and glycolytic activity in tumors may interact in diverse ways [1–3], the dynamics of the tumor microenvironment need to be revealed by future molecular imaging researches.

The over-reductive status is not the only determinant of Cu-ATSM retention in cancer cells because intracellular pH and copper metabolism may also affect the retention of the tracer [32, 33]. However, previous studies, including ours, demonstrated that the uptake of Cu-ATSM correlated with poor patient prognoses in some cancer types, such as HNC, cervical cancer, and rectal cancer [26, 34–36]. Tateishi et al. recently reported that the uptake of ^62^Cu-ATSM was significantly higher in glioblastoma (WHO grade IV) than in lower-grade gliomas [37, 38]. The higher accumulation of ^62^Cu-ATSM may reflect more ROS being produced in tumors as well as a more aggressive tumor phenotype [4–10]. Cu-ATSM PET delineates aggressive or treatment-resistant regions in tumors and this information may potentially be used for intensity-modulated radiation therapy (IMRT) planning and prognostic predictions for patients with these cancer types [39, 40].

**Fig 8:**
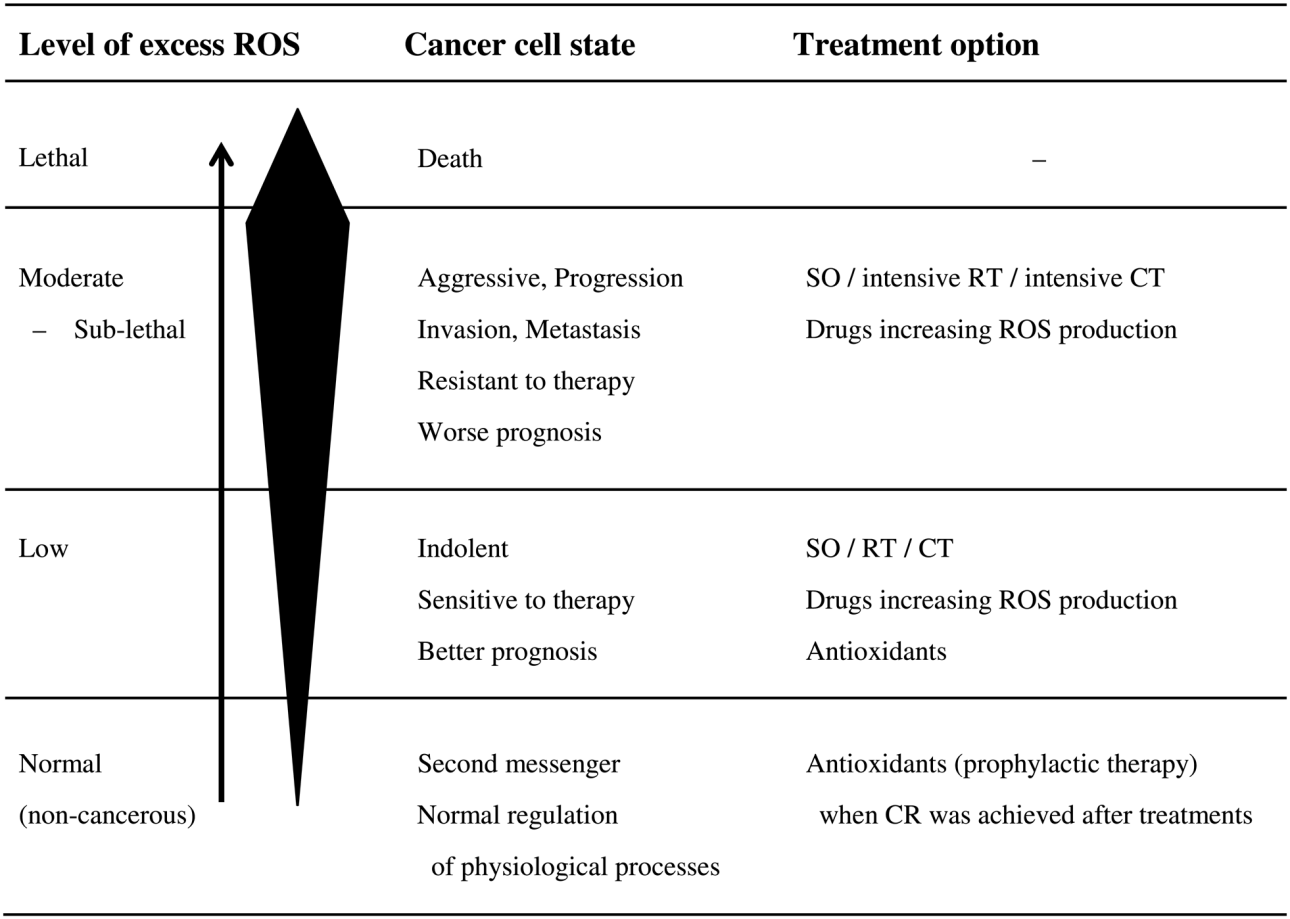
Hypothetical relationships between excess ROS production levels, cancer cell states, and treatment options. The arrow indicates the direction of the level of excess ROS from the normal to lethal range. The black deformed quadrangle represents excess ROS concentrations. SO: surgical operation, RT: radiation therapy, CT: chemotherapy, CR: complete response.

Furthermore, the tumor redox status imaged by Cu-ATSM PET may provide criteria for redox-modulating strategies in cancer patients [41–48]. In non-cancerous cells, ROS play an essential role as second messengers in the normal regulation of various physiological processes. In cancer cells under mitochondrial dysfunction or hypoxia, they also act as signaling molecules to increase aggressiveness/motility/invasion and resistance to treatments, thereby leading to a worse prognosis with an increase in ROS production. On the other hand, lethal concentrations of ROS may trigger cell death pathways against the antioxidant capacity of cancer cells. Redox-modulating strategies to target these biochemical properties of cancer cells will represent a feasible therapeutic approach that may enable therapeutic selectivity and overcome resistance to treatments. Fig 8 shows a hypothetical relationship between excess ROS production levels, cancer cell states, and treatment options. Since the excessive production of ROS induces apoptosis in cancer cells or the progression of cancer growth, these strategies have been divided into two categories: to increase or reduce cellular ROS levels. Increases in intracellular oxidative stress have long been recognized as one of the mechanisms of action of cancer chemotherapy (CT) and RT. Multidisciplinary therapy (the combination of SO, RT, and CT) may effectively induce lethal injuries in cancer cells when combined with drugs that increase the production of ROS [41, 42]. In contrast, antioxidants may effectively prevent tumor progression, particularly in some kinds of cancer patients showing low levels of excess ROS [43–46]. They may also be used prophylactically for patients in remission in order to prevent recurrence or secondary cancer [47, 48].

Since no previous Cu-ATSM PET studies evaluated optimal segmentation methods for delineating over-reductive areas in tumors, four thresholds (40%, 50%, 60%, and 70% SUV_ATSM_) were examined by the ROC analysis in the present study. The threshold of 70% SUV_ATSM_ was selected because it yielded the largest AUCs for predicting disease progression and cancer death. However, no significant difference was observed among the AUCs of the four thresholds. Thresholds of 80% or higher % SUV_ATSM_, which may more accurately predict patient outcomes, need to be examined in future studies. It is important to note that optimal thresholds need to be obtained separately in each site because the volume definition using % thresholds varies with maximum SUV itself, which may differ among institutes using different PET scanners with different crystals, imaging, and reconstruction protocols. Other thresholding techniques using absolute values such as SUVs also need to be evaluated in the future. Furthermore, it is important to note that Cu-ATSM PET detects surplus electrons in some forms of reductants and does not directly reflect the degree and distribution of ROS in cancer tissues. Further fundamental and clinical validation studies are warranted.

*In vivo* mapping of the tumor redox status has been intensively examined in recent years. MRI with redox-sensitive T1 shortening or paramagnetic chemical exchange saturation transfer (CEST) contrast agents are used to evaluate the tumor redox state and its heterogeneity [11, 12]. Although this MR technique enables non-invasive observations of deep tissues, it is still being developed using animal models and requires a high-magnetic field MRI apparatus such as 7 tesla. In contrast, ^62^Cu-ATSM is produced by a ^62^Zn/^62^Cu generator with synthesis kits, which allows tumor redox PET imaging in humans to be performed conveniently on site. Integrated PET/MR scanners will enable the accurate registration and superimposed display of over-reductive areas in tumors detected by Cu-ATSM PET and MR signals from hydrogen protons (^1^H) including information on metabolites by MR spectroscopy. Keshari et al. recently developed hyperpolarized [1-^13^C]dehydroascorbate (^13^C-DHA) as an MR probe to investigate redox changes in prostate cancer [49]. They compared hyperpolarized ^13^C-DHA MR spectroscopic signals with ^18^F-FDG uptake separately obtained by a small-animal PET/CT scanner [50]. Integrated PET/MR imaging will provide the accurate registration of MR redox signals and ^18^F-FDG uptake in the near future.

There were several limitations to the present study due to the small patient population and absence of additional experimental validation. Clinical stages, primary sites, pathological findings, and therapeutic strategies were heterogeneous. Furthermore, the usefulness of ^62^Cu-ATSM PET for the prognosis of HNC was not evaluated through comparisons with conventional prognostic factors such as the tumor size, advanced nodal stage, and HPV status [51, 52]. The total number of patients (*n* = 30) was not sufficient to perform multivariate tests. A larger number of patients and multi-regression analysis are required in future research. In a previous animal study, a time-dependent reversal in the intra-tumoral distribution of ^64^Cu-ATSM was reported in rats bearing homograft prostate tumors [17]. Over-reductive regions may change location in tumors according to cycling hypoxia; however, the time scale is not known. Time-dependent changes in tracer distribution need to be evaluated in humans using ^64^Cu-ATSM labeled with copper-64 (t_1/2_ = 12.7 h), allowing for longer observations. In addition, the stability of ^62^Cu-ATSM complexes in humans has not yet been determined. Comparisons of tumor ^62^Cu radioactivity in patients injected with ^62^Cu-ATSM and ^62^Cu salts (^62^CuCl_2_ or ^62^Cu-acetate) may be needed in order to determine whether ^62^Cu radioactivity in HNC is derived from the tumor uptake of intact ^62^Cu-ATSM complexes or cellular uptake of the ionic ^62^Cu radionuclide that disassociated from ^62^Cu-ATSM [33, 53].

## Conclusions

Pretreatment ^62^Cu-ATSM PET provided clinically relevant information on the tumor redox status that was predictive of outcomes in patients with HNC. The high tumor uptake of ^62^Cu-ATSM may predict resistance to treatments and poor prognoses in patients with HNC. A reductive intensity and reductive tumor burden may both be determinants of HNC patient outcomes and potentially support optimally-individualized treatments for each patient.
